# Editorial: The role of micronutrients in renal physiology and pathophysiology

**DOI:** 10.3389/fphys.2023.1207279

**Published:** 2023-05-10

**Authors:** Deanne H. Hryciw, Hassan Askari, Niels Olsen Saraiva Camara, Shadi K. Gholami, Jamshid Roozbeh

**Affiliations:** ^1^ School of Environment and Science, Griffith University, Nathan, QLD, Australia; ^2^ Griffith Institute for Drug Discovery, Griffith University, Nathan, QLD, Australia; ^3^ Institute of Experimental Medicine and Systems Biology, RWTH Aachen University, Medical Faculty, Aachen, Germany; ^4^ Department of Immunology, Institute of Biomedical Sciences, Universidade de São Paulo, São Paulo, Brazil; ^5^ Division of Endocrinology, Diabetes and Hypertension, Department of Medicine, Brigham and Women’s Hospital, Harvard Medical School, Boston, MA, United States; ^6^ Shiraz Nephro-Urology Research Center, Shiraz University of Medical Sciences, Shiraz, Iran

**Keywords:** nutrient, kidney, diet, pathology, deficiency, overconsumption

Renal function is critical for normal physiology, and perturbations resulting in pathophysiology significantly impact not only renal function but homeostasis in the whole body. Pathophysiology or renal disease is a significant global health Research Topic which impacts healthcare costs to governments and their citizens. Acute and chronic kidney diseases have several factors which contribute to their development and progression, with etiology, diagnosis, prevention, and treatment core to the management of these diseases. Micronutrients, such as vitamins and minerals, play an important role in renal physiology, with deficiency or abundance of micronutrients likely to augment pathology in renal disease.

This Research Topic aims to explore the effects of micronutrients on kidney function and the role micronutrients play in contributing to the disease phenotype. The Research Topic contains 3 review articles, 2 original manuscripts, and 1 systematic review. Collectively, they demonstrate specific micronutrients contribution to renal disease and its progression.

The first review article by Yan et al. focuses on ion channel-targeted therapeutics to perturb the process of renal impairment and fibrosis associated with some kidney diseases. Certainly, there are several ion channels in the kidney contribute to renal fibrosis, potentially modulating a number of downstream signaling cascades. The focus of Yan et al. is on sodium, potassium, chloride, and calcium-mediated pathways controlled by cystic fibrosis transmembrane conductance regulator (CFTR), transmembrane member 16A, calcium-release-activated calcium channel, purinergic receptor, transient receptor potential channels, epithelial sodium channel (ENaC), Na^+^, K^+^ -ATPase, Na^+^ -H^+^ exchangers, and Ca^2+^-activated K^+^ channels, voltage-dependent K^+^ channel, ATP-sensitive K^+^ channels. This extensive review outlines the importance of channel interactions and how these contribute to renal fibrosis. Critical to this discussion, as ion channels are widely expressed in most cells and tissues, drugs that focus on tissue and subtype specificity need to be designed to avoid safety issues. Yan et al. propose a precise intracellular delivery of novel modulators based on nanoplatforms might be a promising option.

In the review by Baltusnikiene et al. these authors summarise the beneficial and adverse effects of vitamin E supplementation on renal function in animal studies as well as humans. Interestingly, the authors suggest that the varied results associated with vitamin E and its effect on the kidney are associated with specific dose effects. The main measurement associated with toxicity, or upper limit of toxicity (UL) for vitamin E varies worldwide. For example, in Europe, the UL for adults is 300 mg per day (EFSA Panel on Dietetic Products, 2015), while in the United States, the UL for adults is 1000 mg per day (Wheldon et al., 1983). In mice studies, higher doses of vitamin E result in tissue toxicity, oxidative stress, and inflammation. Other studies have not supported the adverse outcomes associated with vitamin E usage. More emerging research has identified sex differences in the effects of vitamin E and renal outcomes in humans (Hara et al., 2021). Any potential benefits for vitamin E supplementation on renal health require a clear understanding of *in vivo* outcomes on human health, the sex of the individual, and the interaction of vitamin E with other micronutrients.

The final review manuscript by Goncalves et al. focused on vitamin D and chronic kidney disease (CKD) and the role of crosstalk between tubular epithelial cells and macrophages. Vitamin D deficiency significantly impacts CKD progression through the promotion of inflammation and dysfunction. The relationship between tubular epithelial cells and macrophages is critical as these cells activate vitamin D and express vitamin D receptors. Further, Goncalves et al. discuss how vitamin D modulates lipid metabolism in tubular epithelial cells and macrophages. These authors suggest that vitamin D-mediated cell signalling should be investigated in the future to target CKD progression.

The original research manuscript by Dai et al. investigated the role of the vitamin D receptor (VDR) in male VDR knockout (VDR-KO) and renal proximal tubular specific VDR overexpressing (VDR-OE) mice. In these mice, acute kidney injury (AKI) was induced by lipopolysaccharide (LPS) injection. Dai *et al.* demonstrated that treatment with a vitamin D analogue or VDR-specific overexpression restored glucose metabolism reprogramming and renal injury in the model of AKI, whereas VDR-KO resulted in a more severe glycolytic shift and renal injury. Further, the vitamin D pathway controlled AKI-associated renal inflammation and apoptosis via an AMP-kinase pathway. Thus, Dai et al. suggest that research should investigate vitamin D to alleviate AKI-induced metabolic programming.

The original research manuscript by Xie et al. focused on the relationship between dietary choline, the gut microbiome, and chronic kidney disease-induced cardiac dysfunction. CKD and cardiovascular disease (CVD) are closely linked, with CVD being the primary cause of mortality in patients with CKD (London, 2003). Using male CD1 mice subjected to five-sixths nephrectomy, Xie et al. demonstrated that dietary choline, prior to induction of CKD by nephrectomy, inhibits cardiac angiogenesis by reducing cardiac Hif-1α protein. Further, -gut microbe-generated toxin trimethylamine-N-oxide (TMAO) improved cardiac dysfunction. Thus, TMAO, activated by dietary choline, improves cardiac dysfunction in CKD mice via Hif1α.

The final manuscript, a systematic review, focused on autophagy in calcium oxalate kidney stone formation. Li et al. systematically reviewed the literature using the PRISMA guidelines and identified that in *vitro*, animal, and human studies, upregulation and downregulation of autophagy have the potential to ameliorate injury associated with kidney stones. Importantly autophagy has the potential to interact with downstream signaling pathways. For example, nuclear translocation of the autophagy-related protein TFEB contributed to kidney stone formation (Unno et al., 2020). Li et al. proposed that future therapeutics should investigate combination therapies that target oxidative stress and autophagy in calcium oxalate kidney stone formation.

Collectively the manuscripts in this Research Topic highlight the importance of micronutrients in acute and chronic renal disease. Future studies clearly identifying cell signalling pathways, specific dosage effects, and sex differences, will provide important insight into the role of micronutrient perturbations in renal physiology and pathophysiology.
